# Outlook and opportunities for engineered environments of breast cancer dormancy

**DOI:** 10.1126/sciadv.adl0165

**Published:** 2024-03-08

**Authors:** Nathan R. Richbourg, Ninette Irakoze, Hyuna Kim, Shelly R. Peyton

**Affiliations:** ^1^ Department of Chemical Engineering, University of Massachusetts Amherst, MA 01003, USA.; ^2^ Molecular and Cellular Biology Graduate Program, University of Massachusetts Amherst, MA 01003, USA.; ^3^ Department of Biomedical Engineering, University of Massachusetts Amherst Amherst, MA 01003, USA.

## Abstract

Dormant, disseminated breast cancer cells resist treatment and may relapse into malignant metastases after decades of quiescence. Identifying how and why these dormant breast cancer cells are triggered into outgrowth is a key unsolved step in treating latent, metastatic breast cancer. However, our understanding of breast cancer dormancy in vivo is limited by technical challenges and ethical concerns with triggering the activation of dormant breast cancer. In vitro models avoid many of these challenges by simulating breast cancer dormancy and activation in well-controlled, bench-top conditions, creating opportunities for fundamental insights into breast cancer biology that complement what can be achieved through animal and clinical studies. In this review, we address clinical and preclinical approaches to treating breast cancer dormancy, how precisely controlled artificial environments reveal key interactions that regulate breast cancer dormancy, and how future generations of biomaterials could answer further questions about breast cancer dormancy.

## Teaser

Current approaches to treat dormant cancer are reviewed to provide an outlook for future models of dormancy and relapse.

## INTRODUCTION

Even after initially successful treatment, approximately 40% of early-stage breast cancer patients develop relapse in distant organs, indicating that disseminated tumor cells (DTCs) can remain dormant for many years before growing into a detectable and symptomatic tumor (*1*–*3*). It is difficult to treat these dormant DTCs because they are not actively cycling and thus cannot be killed by traditional chemotherapies. Furthermore, dormant DTCs are dispersed as single cells or small clusters among many other cells and are therefore challenging to clinically detect in situ (i.e., without biopsies). Critically, dormant DTCs extend the threat of metastatic breast cancer, which presents a 30% 5-year survival rate compared to a 99% 5-year survival rate for localized breast cancer (*4*). Therefore, further understanding of how, when, and why DTCs enter and exit dormancy is needed for treating latent metastatic breast cancer. Throughout this review, we specifically address these dormant DTCs, which have distinct detection and treatment challenges from residual disease at the primary site.

**Fig. 1.**
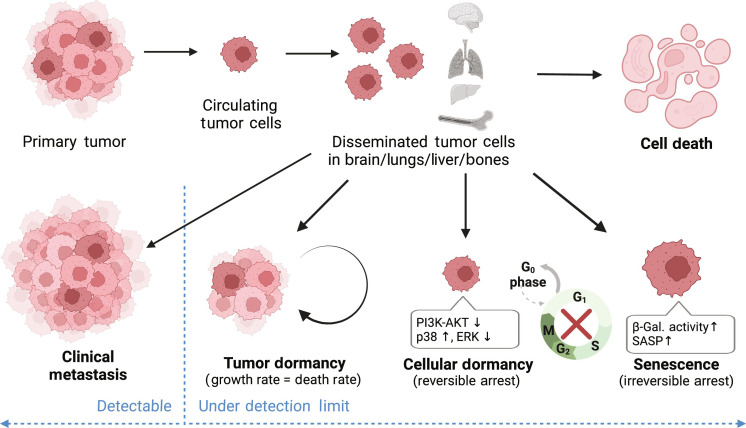
Outcomes of DTCs. Disseminated breast cancer tumor cells may grow into detectable metastases, reach an equilibrium in tumor dormancy, become single-cell dormant or senescent, or die. Both dormancy and senescence are marked by cell cycle arrest, but senescence is associated with increased β-galactosidase (β-gal) activity and expression of a senescence-associated secretory phenotype (SASP), whereas dormancy is associated with reduced expression of PI3K-AKT, an increase in the ratio of phosphorylated p38 to phosphorylated ERK expression, and potential for relapse. Figure created in BioRender.

Determining the potential for relapse in dormant DTCs is a grand challenge in cancer research. One of the pervasive roadblocks in achieving this is that the bulk of the field’s knowledge of tumor and cellular dormancy is limited to static, endpoint measurements in vivo, which cannot capture the critical transitions into and out of dormancy. For instance, immunohistochemistry (IHC) of fixed clinical and in vivo specimens can provide insight into the localization of dormant or proliferating cells within the matrix by comparing the levels of Ki67 expression (*5*, *6*); however, IHC cannot determine whether the observed nonproliferative cells are capable of eventual outgrowth, nor whether the factors from their microenvironment would affect the outgrowth. Furthermore, dormant DTCs are maintained as either independent dormant cells (cellular dormancy) or as small clusters of cells with a balance of proliferation and death (tumor dormancy; Fig. 1) (*7*–*10*). Differences in cell behaviors between these two dormancy paradigms exacerbate issues with analyzing and treating dormancy, such as establishing consistent markers of dormancy (*11*). Common markers of in vivo dormancy include cell cycle reporters and the ratio of phosphorylated p38 to phosphorylated extracellular signal-regulated kinase (ERK), which can be validated for both cellular and tumor dormancy using in vitro models (*12*). However, the reliability of these markers and their ability to distinguish between dormant, senescent, and stem-like DTCs remain controversial. Techniques that analyze the characteristics of single cells instead of population-level averages, such as flow cytometry and single-cell RNA-seq, are critical to distinguishing between cellular and tumor dormancy as well as other DTC phenotypes (*13*). Single-cell methods are especially important for parsing information from coculture models with multiple cell types and can further resolve important interactions such as signaling between senescent and dormant cells.

 In vitro models aim to simulate the environmental triggers that induce and reactivate dormant DTCs, providing both controlled stimuli to create that transition and more opportunities for precisely characterizing how cell-environment interactions affect dormant and reactivated DTCs (*14*–*16*). These in vitro models establish preliminary hypotheses for individual mechanisms of environmental dormancy regulation that can then be validated in vivo and applied in clinical treatment (*17*, *18*). For example, in vitro coculture models with specific stromal cell types identify which stromal cells tend to support cancer cell dormancy, corroborating in vivo studies that observe dormant DTCs colocalizing with those stromal cell types (*19*). However, dynamic transitions within in vitro models are still a relatively new paradigm, and traditional in vitro modeling approaches fail to capture dynamic transitions between dormancy and reactivation (*20*). The next generation of in vitro models for breast cancer dormancy needs to clearly distinguish between cellular and tumor dormancy, reliably simulate long-term dormancy and reactivation, and facilitate robust characterization of cells and cell-environment interactions at all stages of dormancy and reactivation.

Here, we review how control of cell-environment interactions via in vitro model systems has contributed to our understanding and treatment of breast cancer dormancy. We discuss how biomaterials can be designed to creatively study the interface of breast cancer cell biology and the dormant tumor microenvironment (TME). We first describe the state of clinical treatment to kill dormant breast DTCs and survey the current clinical trials and most promising preclinical studies targeting breast cancer dormancy. We then describe the insights gained from in vitro breast cancer dormancy models, acknowledging their successes and limitations. Last, we look forward, prescribing directions for innovating on these model environments, suggesting ways to apply their vast potential toward a more complete understanding of how the microenvironment contributes to dormancy and relapse in metastatic breast cancer.

## PRECLINICAL AND CLINICAL TREATMENT APPROACHES FOR BREAST CANCER DORMANCY

To provide context for the need for in vitro models of breast cancer dormancy, the following sections discuss preclinical and clinical strategies for treating breast cancer dormancy. Proposed approaches to prevent relapse of dormant breast cancer metastases can be broadly classified into three groups: direct killing of dormant cancer cells, awakening dormant cancer cells and subsequently killing them with chemotherapy, or keeping cells in a dormant state indefinitely (*21*). These approaches take advantage of the different pathways involved in breast cancer dormancy to develop potential therapies, including some currently in phase 2 and phase 3 clinical trials. The following sections discuss preclinical and clinical developments in treating breast cancer dormancy.

### Direct killing of dormant cancer cells

**Fig. 2.**
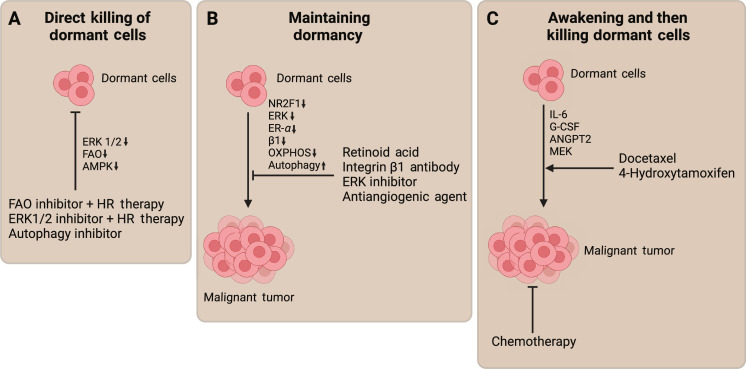
Treatment approaches for breast cancer dormancy. Plausible therapeutic pathway targets to (**A**) directly kill, (**B**) maintain, or (**C**) activate and then kill dormant tumor cells. Figure created in BioRender.

Dormant breast cancer uses several mechanisms to resist typical anticancer treatments, including autophagy-related pathways, adenosine monophosphate–activated protein kinase (AMPK) signaling, and ERK signaling (*22*–*25*). Resistance is often characterized by a reduction in cancer cells’ need for specific pathways required for their survival and growth (*24*, *26*). Dormant cells have a lower metabolic rate than actively dividing cells which reduces their demand for proteins required for cellular growth and division (*27*). Consequently, the efficacy of drugs targeting these proteins may be reduced. Dormant cells can undergo adaptive changes in response to their environment, and these adaptations may involve the down-regulation of certain proteins, allowing the cells to survive in a quiescent state with reduced dependency on typical growth and survival molecular pathways (*18*, *28*, *29*). Preclinical in vitro studies attempt to combat the mechanisms by which these dormant tumor cells evade therapy (*30*). For instance, dormant cells evade anti-estrogen therapies by up-regulating fatty acid oxidation (FAO) and activating the AMPK pathway. In a hypoxic environment, these cells can also resist estrogen receptor (ER)–targeting therapies through the activation of ERK. Inhibition of FAO and ERK improves the efficacy of anti-estrogen drugs in eliminating dormant ER-positive (ER^+^) breast cancer cells (Fig. 2) (*22*, *31*).

NCT04841148 and NCT04523857 are phase 2 clinical trials now recruiting patients with early-stage ER^+^ breast cancer with DTCs in the bone marrow. Both trials leverage preclinical data showing that autophagy, cell cycle, and immune checkpoint pathways play critical roles in tumor dormancy. NCT04841148 tests the effects of hydroxychloroquine, an autophagy inhibitor, or avelumab, a programmed cell death protein 1 and programmed death-ligand 1 (PD-1/PD-L1) inhibitor, alone or combined with palbociclib, a cyclin-dependent kinase 4 and 6 (CDK4/6) inhibitor, for DTC eradication in bone marrow. NCT04523857 investigates hydroxychloroquine alone or in combination with abemaciclib, another CDK4/6 inhibitor.

Other active clinical trials targeting dormant cells involve detecting circulating tumor cell DNA (ctDNA) as a marker of minimal residual disease in patients with breast cancer (NCT03145961, NCT04567420, NCT03285412, and NCT04915755). Upon testing positive for ctDNA after initial treatment, patients in these studies are treated with CDK4/6 inhibitors combined with hormonal therapy, a poly(adenosine-diphosphate–ribose) polymerase (PARP) inhibitor, or a PD-1 inhibitor.

### Sensitizing dormant cancer cells to chemotherapy

Studies show dormant tumor cells resist chemotherapy through slowed or arrested cell cycles and from signals from the microenvironment (*32*, *33*). Restrictive synthetic polymer-based three-dimensional (3D) hydrogels induce dormancy and doxorubicin resistance in MDA-MB-231 cells by reducing drug localization in their nuclei, suggesting that targeting the mechanical properties of the cellular microenvironment could sensitize them to drugs (*34*). In organoid models, dormant D2.0R cells proliferate after docetaxel treatment due to proinflammatory cytokines, interleukin-6 (IL-6) and granulocyte colony-stimulating factor, secreted from stromal cells, revealing the role of microenvironmental cell populations in chemoresistance (*35*).

The microvascular niche also provides an ideal environment for dormant cells to survive chemotherapy. Engineered 3D vascular niches protect dormant ER^+^ breast cancer cells from chemotherapy via integrin-initiated signaling. However, inhibiting integrins β_1_ and α_v_β_3_ sensitizes the dormant cells to chemotherapy (*36*). Notably, this integrin-dependent chemotherapy sensitization is less effective in immune-competent mice than in in vitro models (*37*). Angiopoietin 2 (ANGPT2) is a protein linked to hormone therapy resistance in dormant ER^+^ breast cancer cells. Culturing ER^+^ breast cancer cells in a bone marrow endothelial niche induces dormancy in these cells, and when treated with 4-hydroxytamoxifen, it awakens them through activation of ANGPT2 signaling via integrin β_1_ and Tie2 receptor (*38*).

Targeting stromal cells and integrins and optimizing the timing of chemotherapy administration in conjunction with hormonal therapy may enhance the sensitivity of dormant cells to chemotherapy. Further research into manipulating the microenvironment to reduce dormant cell drug resistance is needed.

### Maintaining dormant states in cells and tumors

Maintaining cancer dormancy can be an attractive therapeutic approach because it proactively reduces the risk of life-threatening overt metastasis, despite requiring ongoing treatment (*39*, *40*). One way of maintaining tumor dormancy is using adaptive therapy, which aims to optimize the treatment regimen based on the tumor’s response to therapy. This involves varying drug administration timing and dosage, and it allows for control of tumor size while minimizing drug resistance development (*41*, *42*).

Nuclear receptor subfamily 2 group F member 1 (NR2F1), a transcriptional regulator, is one of the proteins down-regulated in proliferative breast cancer cells and up-regulated in dormant tumors or cells (*43*, *44*). Targeting this protein by up-regulating it using retinoic acid supports dormancy (*45*). Since low ERK activity is a defining characteristic of dormant cells, inhibition of ERK is another promising strategy for maintaining dormancy (*37*, *46*).

Stiff matrices and redox balancing support chemotherapy resistance in dormant breast cancer cells. Culturing MDA-MB-231s in a high-stiffness (50 kPa) 3D matrix slows their growth, creating a dormant phenotype. The stiff 3D matrix allows the cells to survive oxidative stress caused by Paclitaxel, by activating anti-oxidative signals (*47*). The slow-growing cells regain their ability to proliferate when cultured in a nonrestrictive 3D matrix or immunocompromised rats. This study suggests that targeting oxidative stress responses may keep cells dormant.

Oxidative phosphorylation (OXPHOS) is also a critical pathway in promoting metabolic dormancy across various types of cancer (*48*). Tamoxifen and fulvestrant down-regulate OXPHOS in ER^+^ breast cancer cells, inducing dormancy in vitro and in a mouse model (*49*). However, some cells exit dormancy through the transfer of mitochondrial DNA via extracellular vesicles, which restores OXPHOS. This leads to cell proliferation, thus showing how OXPHOS modulation mediates resistance to hormonal therapies and suggesting that it could be used to maintain cell dormancy.

NCT00195091 is an active phase 2 clinical trial investigating how effectively tetrathiomolybdate treats patients with triple-negative breast cancer (TNBC) at moderate to high risk of recurrence. Tetrathiomolybdate is a copper-chelating drug that mitigates metastasis in some cancers by disrupting angiogenesis (*50*–*53*). Angiogenesis mediates the awakening of dormant cells (*54*), and tetrathiomolybdate could help keep dormant cells quiescent.

### In vitro modeling insight into drug-resistant dormancy

Developing new therapeutic strategies targeting dormant cells and tumors using in vivo models has practical experimental and observational challenges. In vivo models include the interactions between the dormant cells and their natural microenvironment and can evaluate the efficacy of therapies in a whole organism. However, in vivo models are limited by ethical considerations, high cost, low capacity for continuous imaging, and limited ability to manipulate specific pathways (*55*, *56*). These limitations create a need for well-controlled and experimentally accessible in vitro models that closely mimic in vivo systems to find effective therapies. In Table 1, we summarize drug resistance mechanisms found in dormant cells or tumors using in vitro models.

**Table 1.** Chemotherapies and targeted therapies inducing breast cancer dormancy or enabling dormancy escape in in vitro models. Bcl-2, B-cell lymphoma 2 regulator protein; Oct-4, octamer-binding transcription factor 4; Sox-2 SRY-box transcription factor 2; G-CSF, granulocyte colony-stimulating factor; Tie2, tyrosine kinase 2 with immunoglobulin-like and epidermal growth factor homology domains.

Drug name	Type of therapy	Platform used	Mechanism involved	Effect on dormancy	Reference
Docetaxel	Chemotherapy	SUM159 cells (TNBC) on 2D TCPS	–	Induction	(*189*)
Doxorubicin	Chemotherapy	SUM159 cells (TNBC) on 2D TCPS	–	Induction	(*189*)
Paclitaxel	Chemotherapy	MDA-MB-231-Br spheroids on 2D TCPS	Low ERK/p38 activity ratio	Induction	(*190*)
Carboplatin	Chemotherapy	MDA-MB-231 on 2D TCPS and 3D polycaprolactone scaffolds	Increase in cyclin D1, increase in Bcl-2, Oct-4, and Sox-2	Induction	(*191*)
Docetaxel	Chemotherapy	D2.0R in 3D Matrigel with murine endothelial cells and embryonic fibroblasts	Release of IL-6 and G-CSF	Escape	(*35*)
Tamoxifen	Targeted therapy	MCF7 on 2D TCPS	OXPHOS down-regulation	Induction	(*49*)
Fulvestrant	Targeted therapy	MCF7 on 2D TCPS	OXPHOS down-regulation	Induction	(*49*)
4-Hydroxytamoxifen	Targeted therapy	MCF7 in bone marrow endothelial niche on 3D Matrigel	Endothelial Tie2 receptor expression and integrin β_1_ expression	Escape	(*38*)

Overall, using in vitro models provided valuable insights into the mechanisms of chemoresistance and targeted therapy resistance in dormant breast cancer cells. Combined with in vivo models, in vitro models can help find treatment strategies to overcome drug resistance in dormant breast cancer cells and find biomarkers to help with the early detection of metastasis. However, in vitro models still need improvements to reduce false-positive results that fail to translate to in vivo models (*57*). Studies using immune-competent in vivo models yield different drug responses from in vitro models, which is likely due to the complexity of immune signaling that is not viable to fully replicate in vitro (*35*, *36*). Incorporating more of the target tissue’s biochemical aspects into in vitro models would help to reduce the gaps between in vivo and in vitro drug responses. The in vitro drug resistance studies in Table 1 use relatively basic 2D tissue culture polystyrene (TCPS), cell-derived Matrigel, or polycaprolactone 3D scaffolds. While these systems are readily accessible, they do not introduce many of the environmental features relevant to dormant, disseminated breast cancer cells. As discussed in the following section, environmentally instructive in vitro models yield more information about how disseminated breast cancer cells participate in feedback loops with their environment that induce, maintain, and end dormancy. Studies that combine those well-controlled environments with drug resistance experiments reveal the significance of the environment in regulating both dormancy and drug resistance.

## ENVIRONMENTAL CONTROL OF BREAST CANCER DORMANCY IN VITRO

**Fig. 3.**
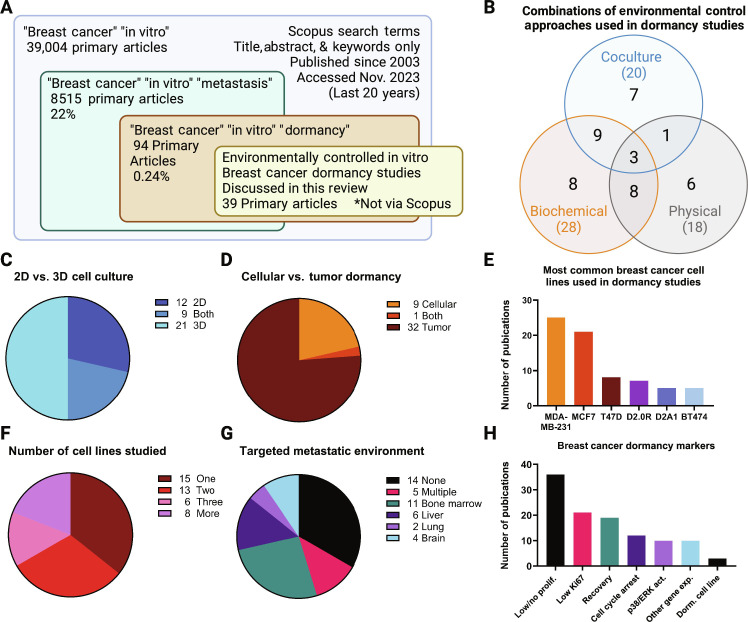
Twenty years of in vitro breast cancer dormancy models at a glance. (**A**) Scopus search terms demonstrate the scarcity of in vitro breast cancer dormancy studies relative to in vitro breast cancer and breast cancer metastasis studies. Thirty-nine research articles using in vitro breast cancer dormancy models were selected for further analysis (**B** to **H**) from a broader search not limited to Scopus search terms. Figure created in BioRender.

In vitro models of breast cancer dormancy enable precise and timely control of the cell-environment interactions hypothesized to support or suppress dormancy. Although rapidly growing, the number of breast cancer dormancy studies is scant compared to the overall number of breast cancer studies. Dormancy is addressed in less than 1% of all in vitro breast cancer research articles published in the past 20 years (Fig. 3A). By comparison, metastasis is addressed in 22% of published in vitro breast cancer papers. Here, we review 42 primary research articles that use in vitro models to investigate cell-environment interactions associated with breast cancer dormancy. Key features of each study are summarized in Table 2. This cohort of studies from the past 20 years describes the emerging field of breast cancer dormancy in vitro modeling. Across these papers, three overarching approaches are used to investigate environmental influences on the dormancy of breast cancer cell lines: cocultures with tissue-specific cells, exposure to specific biochemical cues, and altering the physical properties of the culture environment (Fig. 3B). Often, these approaches were applied combinatorially to explore the synergistic and competitive effects of multiple environmental interactions. The majority of studies used a 3D culture environment (Fig. 3C), and most focused on tumor- or population-level dormancy rather than cellular dormancy (Fig. 3D).

**Table 2.** Environmentally regulated in vitro modeling studies of breast cancer dormancy. Recovery: Return to a proliferative state upon adding a proliferation trigger or removing the dormancy stimulus. Other gene expression: Dormancy marked by gene expression profiles not included in typical proliferation, p38/ERK activity, and cell cycle arrest analysis. Dormant line: An established cell line previously associated with dormancy was used as an inherent indicator of dormancy. FHL2, four-and-a-half LIM domains 2 protein; hFOB, human fetal osteoblasts; hMSC, human mesenchymal stem cells; ECM, extracellular matrix; HA, hyaluronic acid; 4NG1, G1-arrested cells that are tetraploid; IP-10, interferon-γ–inducible protein 10; PEG, polyethylene glycol; EphB6, ephrin receptor B6; CoCl_2_, cobalt(II) chloride; HIF-1-α, hypoxia-inducible factor 1-α; TGF-β1, transforming growth factor–β1; MEK1/2, mitogen-activated protein kinase kinases 1 and 2.

Environmental control approach	Environmental control subgroups	Cellular or tumor dormancy	2D or 3D culture	Breast cancer cell lines	Organotypic target	Dormancy markers	Summary	Reference (year)
Physical	Stiffness	Tumor	3D	MDA-MB-231, MCF7	N/A	Low/no proliferation, recovery, low Ki67, cell cycle arrest, other gene expression	Stiff 3D encapsulation gel increases dormancy via FHL2 and p21 nuclear localization.	(*122*) (2023)
Coculture, biochemical	Indirect coculture, bone marrow cell coculture, individual signaling molecule	Tumor	3D	T47D, BT474	Bone marrow	Low/no proliferation, recovery, low Ki67	Soluble factors from bone marrow niche cells can either promote dormancy (hFOB) or proliferation (hMSC).	(*68*) (2023)
Biochemical, physical	ECM composition, serum deprivation, 2D versus 3D	Cellular	Both	MDA-MB-231, MCF7, D2.0R, D2A1, HCC1954, HCC1143	N/A, bone marrow	Low/no proliferation, recovery, low Ki67, p38/ERK activity	Live cell lineage tracing helps to evaluate cellular dormancy in vitro, including differences in 2D and 3D and different culture environments.	(*59*) (2023)
Physical	Stiffness, suspension, cell spheroid size	Tumor	Both	MDA-MB-231Br, BT474Br3	Brain	Low/no proliferation, low Ki67, p38/ERK activity	Spheroids on soft HA substrates are more dormant than suspended spheroids.	(*130*) (2023)
Physical	Viscoelasticity	Cellular	2D	MCF7	N/A	Cell cycle arrest, low Ki67	Breast cancer cell dormancy and senescence are highly sensitive to changes in substrate viscoelasticity.	(*127*) (2022)
Biochemical	ECM composition	Tumor	3D	MCF7	N/A	Low/no proliferation, recovery, p38/ERK activity	3D encapsulation culture without degradability or integrin-binding sites promotes dormancy in MCF7 cells.	(*98*) (2022)
Coculture, biochemical	Indirect coculture, brain cell coculture, vascularization, ECM composition, serum deprivation	Tumor	3D	MDA-MB-231, T4-2	Brain	Low/no proliferation, low Ki67	Astrocyte-produced laminin-211 supports dormancy of brain metastatic breast cancer cells.	(*80*) (2022)
Biochemical, physical	Serum deprivation, 2D versus 3D	Tumor	Both	D2.0R, D2.A1, ZR-75-1	Liver	Low/no proliferation, recovery, low Ki67, cell cycle arrest, other gene expression	Dormant, nonsenescent breast cancer cells in 3D encapsulation or serum deprivation have a large fraction of 4NG1 cells.	(*106*) (2021)
Coculture, biochemical	Liver cell coculture, individual signaling molecule, serum deprivation	Tumor	3D	MDA-MB-231	Liver	Low/no proliferation	IP-10 indirectly drives activation of breast cancer cells from dormancy via signaling from cocultured hepatocytes.	(*78*) (2021)
Biochemical, physical	Serum deprivation, confinement	Cellular	3D	MDA-MB-231, MCF7, MDA-MB-468	N/A	Recovery, low Ki67, p38/ERK activity, cell cycle arrest, other gene expression	Single-cell encapsulation in an agarose coating and silica-PEG gel causes breast cancer cell dormancy.	(*107*) (2021)
Biochemical	ECM composition	Both	3D	MDA-MB-231, BoM-1833, LM2-4175, BrM2A-831	N/A, bone marrow, lung, brain	Low/no proliferation, recovery, p38/ERK activity	In specific hydrogel formulations, different organotropic breast cancer cell lines enter into tumor-level or cellular dormancy states.	(*99*) (2021)
Coculture, biochemical	Lung cell coculture, individual signaling molecule	Tumor	2D	D2.0R	Lung	Dormant line, other gene expression	EphB6 supports the survival of dormant breast cancer cells in the lung.	(*73*) (2021)
Coculture	Lung cell coculture	Tumor	2D	MCF7, D2.0R, T47D-DBM	Lung	Dormant line, other gene expression	RNA-seq analysis of lung cell coculture with breast cancer cells reveals an autophagy-independent lysosomal mechanism of dormant survival.	(*72*) (2021)
Coculture	Indirect coculture, bone marrow coculture	Cellular	2D	MDA-MB-231, MDA-MB-468, T47D	Bone marrow	Cell cycle arrest, other gene expression	MSC extracellular vesicle secretome is primed by breast cancer cells, altering the potential for dormancy of breast cancer cells that receive the extracellular vesicles.	(*83*) (2021)
Biochemical	ECM composition	Tumor	3D	MDA-MB-231	N/A	Low/no proliferation, recovery	A dormancy-inducing environment promotes cellular resistance to doxorubicin.	(*34*) (2020)
Physical	Stiffness, suspension, cell spheroid size	Tumor	Both	MDA-MB-231BR	Brain	Low/no proliferation, recovery, low Ki67	Hyaluronic hydrogel stiffness and cell cluster size determine breast cancer dormancy versus proliferation.	(*120*) (2020)
Biochemical, physical	ECM composition, network organization	Tumor	3D	MDA-MB-231, T47D, BT474	Bone marrow, lung	Low/no proliferation, recovery, other gene expression	T47D ER^+^ breast cancer cells are more dormancy-capable than MDA-MB-231 TNBC cells, with dormancy also affected by the encapsulating hydrogel properties.	(*60*) (2020)
Coculture, biochemical, physical	Indirect coculture, ECM composition, serum deprivation, network organization	Tumor	2D	23 Human cell lines*	N/A, bone marrow	Low/no proliferation, recovery, low Ki67, cell cycle arrest	Producing and organizing fibronectin helps breast cancer cells to survive serum deprivation–induced dormancy in vitro.	(*23*) (2020)
Physical	Stiffness, cell seeding density	Tumor	2D	MDA-MB-231BR, BT474BR3	Brain	Low/no proliferation, low Ki67, cell cycle arrest, other gene expression	Softer hyaluronic acid substrates support breast cancer dormancy, disrupted by higher cell seeding densities.	(*121*) (2020)
Biochemical	ECM composition	Tumor	3D	MDA-MB-231	N/A	Low/no proliferation, recovery	Changing integrin-binding site density and network structure yields different balances of dormancy and proliferation for breast cancer cells.	(*100*) (2019)
Coculture, biochemical, physical	Bone marrow cell coculture, vascularization, individual signaling molecule, 2D versus 3D	Tumor	Both	MDA-MB-231, BoM-1833	Bone marrow	Low/no proliferation, p38/ERK activity	3D coculture with endothelial cells, bone marrow stromal cells, and fetal osteoblasts kept MDA-MB-231 cells dormant but not bone-tropic metastatic variant BoM-1833.	(*69*) (2019)
Coculture	Indirect coculture	Tumor	Both	MCF7	Bone marrow	Low/no proliferation, low Ki67, other gene expression	MSC-derived extracellular vesicles promote a slightly less active phenotype in MCF7s.	(*82*) (2018)
Biochemical, physical	Individual signaling molecule, hypoxia, 2D versus 3D, suspension	Tumor	Both	MDA-MB-231, MCF7	N/A	Low/no proliferation, recovery, low Ki67, cell cycle arrest	CoCl_2_ mimics hypoxia by stabilizing HIF-1-α, resulting in comparable induction of dormancy in MCF7 and MDA-MB-231 cells as true hypoxia.	(*105*) (2018)
Coculture, biochemical	Bone marrow cell coculture, ECM composition, individual signaling molecule	Cellular	2D	MCF7	Bone marrow	Low/no proliferation, recovery	Inflammatory cytokines IL-6, IL-8, and TGF-β1 induce proliferation from dormant breast cancer cells.	(*95*) (2018)
Coculture, biochemical	Liver cell coculture, individual signaling molecule	Tumor	3D	MDA-MB-231	Liver	Low/no proliferation, recovery	Proteomic analysis identifies candidate biomarkers for dormant breast cancer cells in the liver.	(*76*) (2018)
Coculture, biochemical, physical	Indirect coculture, liver cell coculture, individual signaling molecule, serum deprivation, stiffness	Tumor	Both	MDA-MB-231, MCF7	Liver	Low/no proliferation	Activated hepatic stellate cells express high levels of IL-8 that activate dormant breast cancer cells in the liver.	(*79*) (2018)
Biochemical, physical	Serum deprivation, confinement	Cellular	3D	MDA-MB-231, MCF7, MDA-MB-468, MCF10DCIS.COM	N/A	Recovery, low Ki67	Nondegradable cell encapsulation induces dormancy.	(*108*) (2017)
Coculture	Vascularization	Tumor	3D	MDA-MB-231	N/A	Low/no proliferation, p38/ERK activity	Coculturing MDA-MB-231s with endothelial cells in a hyaluronic acid hydrogel increases dormancy markers.	(*81*) (2017)
Biochemical	Hypoxia	Tumor	2D	MDA-MB-231, MCF7, MDA-MB-468, T47D	N/A	Low/no proliferation, recovery, p38/ERK activity, cell cycle arrest	Hypoxia induces cancer stem cell–like dormancy in hypoxia-surviving MDA-MB-231s.	(*74*) (2017)
Coculture, physical	Liver cell coculture, stiffness	Tumor	3D	MDA-MB-231	Liver	Low/no proliferation, low Ki67	The liver microphysiological system has different effects on breast cancer cell dormancy with a polystyrene scaffold or a hydrogel scaffold.	(*77*) (2017)
Coculture	Indirect coculture, bone marrow cell coculture	Tumor	2D	MDA-MB-231, MCF7, T47D, BT474	Bone marrow	Low/no proliferation, p38/ERK activity	Conditioned media from specific bone marrow stromal cell types induce breast cancer dormancy.	(*71*) (2017)
Physical	Stiffness	Tumor	3D	MDA-MB-231	N/A	Low/no proliferation, recovery, low Ki67	Encapsulation in stiff collagen gels promotes dormancy.	(*47*) (2017)
Coculture, biochemical	Bone marrow cell coculture, individual signaling molecule	Tumor	3D	MCF7, MDA-MB-231BRMS1	Bone marrow	Low/no proliferation	Dormant breast cancer cells in a bone cell coculture environment can be stimulated to proliferate by bone remodeling cytokines.	(*1*) (2015)
Coculture	Liver cell coculture	Tumor	3D	MDA-MB-231, MCF7	Liver	Low/no proliferation, low Ki67, cell cycle arrest	Liver niche cells promote breast cancer dormancy.	(*75*) (2014)
Biochemical, physical	ECM composition, individual signaling molecule, 2D versus 3D	Tumor	Both	D2.0R, D2A1	N/A	Low/no proliferation, low Ki67, cell cycle arrest	Src family kinase inhibition keeps breast cancer cells dormant, and coinhibition of MEK1/2 causes dormant cell apoptosis.	(*45*) (2014)
Coculture	Indirect coculture, bone marrow cell coculture, vascularization	Tumor	3D	MDA-MB-231, MCF7, BT474, T47D, SUM159, SUM149, MDA-MB-435, ZR-75-1, LM2-4175, BoM-1833	Bone marrow	Low/no proliferation, recovery, low Ki67, other gene expression	3D coculture with bone marrow cells can inhibit or support breast cancer cell proliferation.	(*70*) (2013)
Coculture, biochemical	Vascularization	Tumor	3D	T4-2	Bone marrow, lung	Low/no proliferation, low Ki67	Mature endothelial cell–derived thrombospondin-1 supports breast cancer dormancy.	(*5*) (2013)
Biochemical	ECM composition	Tumor	3D	D2.0R, D2A1	N/A	Low/no proliferation, dormant line	Fibrotic enrichment of collagen I drives the transition from breast cancer dormancy to proliferation.	(*37*) (2010)
Biochemical	ECM composition, individual signaling molecule	Cellular	2D	MCF7	Bone marrow	Low/no proliferation	Basic fibroblast growth factor initiates two independent pathways to promote breast cancer cell dormancy.	(*94*) (2009)
Biochemical, physical	ECM composition, network organization	Tumor	3D	MDA-MB-231, MCF7, D2.0R, D2A1, 4T1	N/A	Low/no proliferation, low Ki67, cell cycle arrest	Integrin binding to produced fibronectin helps breast cancer cells to begin proliferating from a dormant state.	(*91*) (2008)
Biochemical	ECM composition, individual signaling molecule	Cellular	2D	MCF7, T47D	Bone marrow	Low/no proliferation, p38/ERK activity	Flavopiridol disrupts the fibronectin-dependent pathway of dormant breast cancer cell survival.	(*93*) (2005)
Coculture, biochemical	Bone marrow cell coculture, ECM composition, individual signaling molecule	Cellular	2D	MDA-MB-231, MCF7, T47D	Bone marrow	Low/no proliferation	Basic fibroblast growth factor and fibronectin support a dormant breast cancer cell population.	(*92*) (2004)

*(*23*) initially compared 23 human breast cancer cell lines, including MDA-MB-231 and MCF7 for their ability to survive serum deprivation–induced dormancy before focusing on the behavior of HCC1954 cells.

All 42 studies use breast cancer cell lines instead of primary patient-derived cells or organoids. Most breast cancer cell lines were developed on the basis of their ability to proliferate and have been maintained in culture for decades and therefore may not be an appropriate approximation of naturally occurring dormant cells. The exclusive use of cell lines in these in vitro studies is a major caveat to their relevance to clinical dormancy. However, the intuitive alternative of using primary patient-derived samples is currently not feasible due to the difficulties and uncertain medical value of identifying and extracting dormant breast cancer cells from patients. Relying on cell lines for dormancy experiments is a first-approximation approach reflecting the relatively early stage of in vitro breast cancer dormancy studies, and improving our understanding of dormancy through these preliminary studies will help to develop better criteria and methods for identifying and culturing dormancy-relevant cells.

The most common breast cancer cell line used in in vitro dormancy models is the triple-negative MDA-MB-231, followed by the luminal cell line MCF7 (Fig. 3E). These two cell lines may not be ideal for dormancy studies since MDA-MB-231s are an especially aggressive cell line unlikely to sustain long-term dormancy, and MCF7 is nonmetastatic (*58*). D2.0R is a well-established dormancy-capable murine breast cancer cell line that is often compared to D2A1, a proliferative cell line derived from that same source as the D2.0R cells. Overall, most of the studies investigated three or fewer breast cancer cell lines (Fig. 3F), despite results indicating that dormancy-related cell-environment interactions vary substantially depending on the cell line (*59*, *60*). One notable exception, Barney *et al.* (*23*) demonstrated that only 5 of 23 breast cancer cell lines can maintain a dormant phenotype under extended in vitro serum deprivation conditions. Such broad screening methods help to identify both relevant mechanisms of breast cancer dormancy and capable cell lines for studying dormancy.

 In vitro dormancy studies either focused on a specific organ associated with breast cancer metastasis, compared several organotypic environments, or used a more general biomaterial to investigate broadly applicable cell-environment interactions (Fig. 3G). Tissue-specific models included bone marrow, liver, lung, and brain, matching the four most common sites of breast cancer metastasis (*61*). Furthermore, the vast majority of clinical breast cancer dormancy is found in the bone, matching the relatively high number of bone marrow–targeting studies (*62*, *63*).

Reproducible, quantitative dormancy markers are necessary to validate dormancy-capable cell lines and in vitro breast cancer dormancy models. Across the studies reviewed here, the most consistently measured marker for dormancy is low or no cellular proliferation within the observation period (Fig. 3H). However, the timescale used to confirm dormancy varies dramatically, from 48 hours (*59*) up to 12 weeks (*23*). These study periods dramatically underrepresent clinical dormancy, which can relapse after 10 or even 20 years (*64*). In vitro models of accelerated aging, such as artificial exposure to reactive oxygen species, could bridge those disparate timescales. In addition, the method of proliferation measurement varies from study to study, including total fluorescence of live-tagged breast cancer cells, change in cell cluster size over time, 5-Ethynyl-2'-deoxyuridine (EdU^+^) cell fraction, and cell quantification and metabolic assays. The method for measuring proliferation affects whether dormancy can be interpreted as cellular or tumor-level since population-level stagnation could be a result of balanced growth and death rates (tumor dormancy) instead of individual cell cycle arrest (cellular dormancy). To confirm that the dormant cells are not senescent, nearly half of the studies validated that the dormant cells recovered their proliferative behavior upon removal from a dormancy-inducing environment or stimulus (see Table 2). Low or no nuclear expression of Ki67, indicating cell cycle arrest and nonproliferation, is another commonly measured marker for breast cancer dormancy, evaluated in 19 of the studies. Other methods of validating cell cycle arrest, including flow cytometry cell cycle analysis, fluorescence ubiquitination cell cycle indicators, p21 and p27 nuclear localization and expression, and p16 expression, were used less frequently. The ratio of p38 and ERK activity, primarily measured via Western blots of phosphorylated p38 and phosphorylated ERK (sometimes including qualifying analysis of nonphosphorylated p38 and ERK), was measured as a marker of dormancy in several studies. In general, a high ratio of active p38 to active ERK was associated with dormancy, although this relationship was not consistent across all reports. Methods for correlating gene expression and dormancy, such as an RNA sequencing (RNA-seq)–based dormancy score (*60*), aim to identify trends and nuances in dormant phenotypes that cannot be captured by individual dormancy markers. However, since these methods are unsupervised, they require additional validation in vivo and in vitro to draw robust conclusions about dormancy. Three studies used the prior validation of D2.0R as a dormant cell line as their primary indicator of dormancy.

Collectively, the diversity and inconsistency of methods used in these studies suggest that in vitro breast cancer dormancy should be validated by multiple methods including sustained low or no proliferation followed by a demonstration of proliferative recovery coupled with mechanistic markers of nonsenescent cell cycle arrest. Further studies should investigate the physiological relevance of different dormancy induction methods (e.g., is serum deprivation–based dormancy functionally equivalent to coculture-induced dormancy?) and determine the clinical relevance of different timespans of in vitro–measured dormancy. Well-established (Ki67 and p38/ERK) and developing (RNA-seq analysis) dormancy markers should be cross-evaluated across cell lines and diverse dormancy-inducing environments, in both cellular and tumor dormancy regimes, to evaluate how reliably they relate to clinically relevant breast cancer dormancy.

In the following sections, we expand on the insights gained from the three major approaches to environmentally controlling breast cancer dormancy in vitro: coculture, biochemical cues, and physical contributions. The final section will discuss engineering opportunities for further developing our understanding of cell-environment interactions in breast cancer dormancy.

### Coculture dormancy models

Coculturing breast cancer cells with the specific cell populations that coexist within their metastatic niches may support a dormant breast cancer phenotype (*19*, *65*). First, organotypic models of bone marrow, lung, liver, and brain dominate these dormancy coculture models, which are the preferential sites of breast cancer metastasis. Organotypic models isolate the signaling between local stromal cells and DTCs that enable breast cancer dormancy. Second, blood vessels and the basement membrane proteins surrounding vessels are key promoters of cellular dormancy (*66*), which has motivated the adoption of endothelial cell coculture models. Third, indirect coculture systems using Transwells, extracellular vesicles, or conditioned media allow for the identification of paracrine and extracellular signaling pathways critical for regulating breast cancer dormancy (*67*).

The skeleton is the most common clinical site of breast cancer dormancy, and several models containing bone marrow cell cocultures have been used to study how different bone marrow stromal cells promote proliferation or dormancy of breast cancer cells. Human fetal osteoblasts (hFOBs) promote breast cancer dormancy (*68*–*71*), whereas mesenchymal stem cells (MSCs) support proliferative tumors (*68*, *70*). The bone marrow fibroblast cell line HS5 also promotes dormancy (*71*) and has been used in combination with hFOBs and human umbilical vein endothelial cells (HUVECs) as a breast cancer dormancy niche (*69*, *70*). Critically, this dormancy niche restricts MDA-MB-231 proliferation but not the proliferation of the bone metastatic MDA-MB-231–derived cell line BoM-1833, suggesting that bone metastatic breast cancer has intrinsic resistance to the proliferation-inhibiting signals of the bone marrow niche (*69*, *70*).

Coculture with the lung alveolar stromal cell line TT1 down-regulated proliferative RNA-seq signatures in the murine dormancy–associated breast cancer cell line D2.0R (*72*). Mechanistic analysis of this system indicates a lysosome-dependent, autophagy-independent method of dormant breast cancer cell survival in the lungs mediated by the ephrin receptor EphB6 (*72*, *73*). This lysosome-dependent dormancy survival mechanism in a lung-mimicking coculture contrasts autophagy-dependent dormancy survival mechanisms in hypoxic (*74*) and bone marrow–mimetic environments (*60*, *68*).

The Wells lab used a liver microphysiological system (MPS) to investigate breast cancer dormancy in the liver, coculturing breast cancer cells with combinations of hepatocytes, nonparenchymal cells, and hepatic stellate cells (*75*–*79*). Coculture with hepatocytes and nonparenchymal cells in the liver MPS promotes breast cancer cell dormancy (*75*), but coculture with activated stellate hepatocytes promotes breast cancer cell proliferation in the liver MPS. For MDA-MB-231 cells, their reactivation is linked to the soluble signal IL-8 highly expressed by hepatic stellate cells, but MCF7 cells do not respond to IL-8, indicating distinct, cell line–dependent mechanisms of reactivation from dormancy (*79*). Furthermore, interferon-γ–inducible protein 10 (IP-10) stimulates MDA-MB-231 cells to leave dormancy in the hepatocyte-containing liver MPS but has a negligible effect on MDA-MB-231 dormancy without hepatocytes, indicating an indirect reactivation mechanism dependent on hepatocyte interactions.

A single study by Dai *et al.* (*80*) has investigated brain cell coculture for breast cancer dormancy in vitro. This study used combinations of human brain fibroblasts (HBAFs), HUVECs, and astrocytes, finding that all three cell types are required to support dormant culture of T4-2 or MDA-MB-231 breast cancer cells. Stimulated cellular overexpression of extracellular matrix (ECM) components indicated that astrocyte-produced laminin-211 is critical in regulating breast cancer cell dormancy in a brain microenvironment.

The ubiquitous role of vascularization in tumor development and its presence across tissues make vascularized endothelial cell cocultures an important method for studying breast cancer dormancy. A seminal study by Ghajar *et al.* (*5*) identified mature vasculature as a supporter of breast cancer dormancy, whereas neovasculature supports breast cancer cell proliferation. Mature vasculature highly expresses thrombospondin-1, which promotes breast cancer cell dormancy even in the absence of endothelial cells. However, a following study by Ghajar and colleagues (*80*) qualified that for the same breast cancer cell line (T4-2), a combination of HBAFs, HUVECs, and astrocytes was needed to maintain dormancy, and mature vascularization was insufficient alone. Independently, Kassim *et al.* (*81*) found that coculturing MDA-MB-231s with endothelial cells decreased cancer cell proliferation and increased the p38/ERK activity ratio, suggesting some extent of dormancy induction. Two bone marrow–mimicking studies incorporated endothelial cells as components of the dormancy-supporting niche but did not isolate the influence of the endothelial cells on breast cancer dormancy (*69*, *70*). Together, these studies indicate that the role of vascularization on breast cancer dormancy may depend on the maturity of the endothelial cells and vessels as well as interactions with tissue-specific stromal cells.

In addition to cocultures organized by cell types, indirect coculture approaches distinguish mechanisms of cell-cell communication and make it easier to analyze breast cancer cell populations that are physically separate from other cells. Transwell and conditioned media studies indicate whether niche cells facilitate dormancy via soluble molecule paracrine signaling. Indirect Transwell culture with hFOBs promotes dormancy while Transwell MSCs promote proliferation, and exchanging hFOB and MSC Transwells switches the dormancy and proliferation effect (*68*). Hepatic stellate cells promote breast cancer cell proliferation from dormant conditions via Transwells (*79*). Conditioned media studies demonstrate that indirect cell-cell communication is sufficient for HS5 and hFOB-mediated dormancy, complementing the results from the hFOB Transwell study (*70*, *71*) but insufficient for astrocyte-mediated dormancy (*80*). MSC-derived extracellular vesicles contribute to a dormant phenotype for breast cancer cells (*82*, *83*). Together, these studies highlight examples of cell-secreted soluble molecules inducing dormancy or proliferation as well as situations where coculture must be direct to induce dormancy, suggesting multiple mechanisms for coculture regulation of breast cancer dormancy. Furthermore, the reversible dormancy/proliferation switch dependent on the indirect signaling from different bone marrow niche cells demonstrates the complexity of breast cancer dormancy in bone marrow and the importance of specific signaling niches within bone marrow.

Coculture is a powerful tool for investigating breast cancer dormancy in vitro, but applying biochemical modifications (e.g., additional signaling molecule stimulation) and physical alterations (e.g., 3D encapsulation) helps to further interpret the mechanisms of cell-cell interactions. Future research should expand on these models, especially developing lung and brain cell cocultures to match the detailed studies in bone marrow and liver. Further studies in vasculature interactions with different tissue-specific cells and indirect coculture methods will provide a nuanced understanding of cell-cell interactions that cannot be achieved in vivo.

### Biochemical environmental signaling

Breast cancer dormancy models may use specific biochemical environmental signals to influence cell behavior without the added complexity of including secondary cell types. These biochemical models parse signaling mechanisms from multifaceted cell-cell interactions. The ECM provides many biochemical signals to DTCs, including cell adhesion sites (*84*–*87*), matrix metalloproteinase (MMP)–degradable motifs (*88*), and growth factor–immobilizing regions (*89*). Even without ECM interactions, signaling molecules in the microenvironment are often key determinants of breast cancer cell dormancy (*90*). Specific cytokines and growth factors drive cancer dormancy or proliferation, and deprivation of serum or oxygen (hypoxia) induces dormancy. In vitro studies using biochemical factors to investigate breast cancer dormancy are discussed below.

Cell adhesion to specific ECM proteins affects dormancy outcomes. D2.0R cells cultured in basement membrane extract are activated from dormancy by the addition of type I collagen unless Src is inhibited, restricting collagen I’s ability to trigger phosphorylation of focal adhesion kinase (*37*, *45*). Adding fibronectin to the basement membrane extract enabled proliferation of D2.0R cells (*91*). However, the combination of basic fibroblast growth factor 2 (FGF-2) and a fibronectin substrate mechanistically supports dormancy of MCF7 cells but not MDA-MB-231 cells (*92*–*95*). In addition, cell-reorganized fibronectin is necessary for survival during serum deprivation–induced dormancy (*23*). These seemingly conflicting results regarding the role of fibronectin in breast cancer dormancy may emerge from the functions of different isoforms and conformations of fibronectin (*96*, *97*).

Slater and colleagues created a polyethylene glycol (PEG) hydrogel–based model that primarily manipulates MMP degradability and integrin-binding site [arginine-glycine-aspartic acid (RGD)] concentration to assess how those two biochemical environmental components affect breast cancer cell dormancy and proliferation (*34*, *98*–*100*). These studies identified combinations of degradability and integrin-binding site availability that facilitated cell growth, balanced single-cell dormancy, balanced tumor cluster dormancy, or minimal single-cell survival, dependent on the hydrogel formulation and cell line: MDA-MB-231, bone-tropic BoM-1833, lung-tropic LM2-4175, or brain-tropic BrM2A-831 (*99*).

Several in vitro biomaterial-driven studies cross-evaluated the comparative effects of multiple ECM compositions on breast cancer dormancy. Barney *et al.* (*23*) used serum deprivation to induce dormancy of breast cancer cells cultured on TCPS, collagen, and selected bone marrow proteins before further investigating fibronectin and decellularized ECM. Kim *et al.* (*59*) extended this approach, combining serum deprivation with 2D and 3D environments of collagen, Matrigel, or integrin-binding peptides. Using live-cell lineage tracking over 96 hours, Kim *et al.* identified that 3D Matrigel supported a large population of dormant D2.0R cells and 3D collagen contained more cells with senescent behaviors, whereas D2A1 cells in both environments had more cell death. Ovadia *et al.* (*60*) created basement membrane– and bone marrow–mimicking hydrogels, differentiated by the integrin-binding peptide incorporated (IKVAV and GFOGER, respectively), finding differences in the RNA-seq–based dormancy scores based on the different environments.

The list of individual soluble signaling molecules associated with breast cancer dormancy is extensive and overlaps with several well-established immune signaling pathways and tissue-specific feedback loops. As discussed above, FGF-2 coordinates with fibronectin to support breast cancer dormancy (*92*–*94*), but adding IL-6, IL-8, or transforming growth factor β1 (TGF-β1) to that system disrupts dormancy, providing evidence toward inflammation-stimulated reactivation from dormancy in bone marrow (*95*). IL-8 also activates dormant breast cancer cells in the liver (*79*). Thrombospondin-1 supports breast cancer dormancy even when delivered without endothelial cell coculture, strongly supporting a soluble signaling pathway (*5*). Sosnoski *et al.* (*1*) reported that tumor necrosis factor–α (TNF-α) drives breast cancer proliferation in a bone marrow–mimicking coculture independently or co-applied with IL-β1. Contrastingly, Pradhan *et al.* (*68*) demonstrated that TNF-α and monocyte chemoattractant protein 1 independently and cooperatively support breast cancer dormancy, also in a bone marrow–mimicking coculture. The different outcomes may be associated with the different coculture cell types, breast cancer cell lines, or different TNF-α–mediated signaling pathways. These studies highlight that individual signaling molecules that have a notable role in dormancy may produce different outcomes depending on other interactions within the microenvironment.

Hypoxia is a cell-instructive lack of oxygen that commonly occurs in the cores of large, poorly vascularized tumors (*101*), as well as in bone marrow hematopoietic stem cell niches (*102*), which may also support dormant DTCs (*103*, *104*). Two in vitro models have investigated the connection between hypoxia and breast cancer cell dormancy (*74*, *105*). Lee *et al.* (*105*) used cobalt dichloride (CoCl_2_) to stabilize hypoxia-inducible factor 1-α (HIF-1-α), demonstrating that the CoCl_2_ treatment was functionally equivalent to true oxygen deprivation in inducing dormancy and subsequent recovery for MCF7 cells with 4 days of treatment. Carcereri de Prati *et al.* (*74*) used a more stringent method of oxygen deprivation, subjecting cells to 3 cycles of 7 days of hypoxia and 7 days of normoxic recovery, thereby isolating a population of MDA-MB-231 cells capable of surviving for 3 months under hypoxia. By their metrics, MDA-MB-231 cells could survive hypoxia and recover proliferation, but MCF7, T47D, and MDA-MB-468 cell lines could not. These studies reflect the tension between the convenience of short-term in vitro studies and the physiological relevance of longer-term studies. Is long-term hypoxia a critical factor in bone marrow–mediated dormancy of breast cancer cells?

Serum deprivation is used as an effective in vitro method of establishing dormant breast cancer cell cultures. Barney *et al.* (*23*) used serum deprivation for up to 12 weeks to screen the dormancy capability of 23 breast cancer cell lines, identifying fibronectin reorganization as a mechanism of survival under serum deprivation–induced dormancy. Serum-deprived ZR-75-1 and D2.0R breast cancer cells are capable of recovering proliferation and use dormancy-associated intracellular signaling pathways and not senescence-associated pathways (such as accumulation of DNA damage), further validating the use of serum deprivation as a dormancy-inducing method (*106*). In addition to population-scale serum deprivation–induced dormancy, serum deprivation with live-cell lineage tracing identifies single cells capable of cellular dormancy, possibly facilitating downstream isolation of individual dormancy-capable breast cancer cells (*59*). Two studies by Preciado *et al.* (*107*, *108*) used serum deprivation as a positive control comparison for single-cell encapsulation-based dormancy, further validating serum deprivation–based dormancy. The physiological relevance of serum deprivation–based dormancy depends on whether there are natural conditions for extended serum deprivation in breast cancer metastatic sites and whether the molecular mechanisms for serum deprivation–induced dormancy correspond with natural dormancy.

Several studies co-applied serum deprivation with another stimulus to establish breast cancer dormancy. Dai *et al.* (*80*) and Ghajar *et al.* (*5*) both used serum deprivation during the breast cancer cell culture period of their coculture systems to avoid exogenous factors masking the effects of endothelial cell–derived angiocrine factors on tumor growth. Clark *et al.* (*78*) and Khazali *et al.* (*79*) used serum-free media in conjunction with hepatocyte coculture in a liver MPS to support breast cancer cell dormancy. The effects of niche cell coculture on breast cancer dormancy observed in these studies are complicated by the influence of serum deprivation on dormancy, which could either be confounding or complementing the coculture dormancy signaling unless intermediate studies with coculture and serum-rich media are included to clarify.

Biochemical components of the environment, including insoluble ECM components and soluble signaling molecules (or their absence), contribute to the regulation of breast cancer dormancy in vitro. The effects of adding a single ECM component, such as fibronectin or an IKVAV peptide, on dormancy are context specific and ultimately fail to capture the biochemical complexity of the dormant cancer microenvironment. Tissue-specific ECM patterns may better capture how biochemical signals balance dormancy, especially in the complex bone marrow microenvironment (*87*). Furthermore, in vitro coculture and individual signaling molecule–based models of breast cancer dormancy provide important tools that can be combined to investigate the physiologically relevant limitations of using serum deprivation to induce dormancy. Last, ECM contributions and solute transport through the ECM are not simply biochemical influences; they are also linked to the physical properties of the dormant microenvironment.

### Physical environmental factors

In vitro models provide control of environmental physical properties that cannot be achieved in vivo, allowing further study of the cell-environment interactions supporting or restricting breast cancer dormancy. Common physical factors studied in vitro that could affect dormancy include 2D substrate versus 3D encapsulation culture (*109*, *110*), substrate or matrix stiffness and viscoelasticity (*111*, *112*), cell confinement or suspension (*113*), network organization and anisotropy (*110*, *114*). and distances between individual cells and/or size of cell clusters or spheroids (*115*). The influence of environment-restricted solute transport on cell proliferation is not well-investigated but is likely to have a strong effect when cells are encapsulated in 3D hydrogels (*116*, *117*). The interconnectedness and combinations of these physical environmental factors provide rich cell-instructive environments that complement signaling from other cells and biochemical signaling components. Below, we address how these physical factors affect breast cancer dormancy in controlled in vitro models.

Culture dimensionality (2D versus 3D) affects how cells sense their environment, with 3D environments more accurately recreating in vivo conditions for breast cancer dormancy applications. Breast cancer cellular dormancy rates are increased in 3D collagen and Matrigel environments compared to equivalent 2D substrates, with both conditions subject to serum deprivation (*59*). While D2.0R cells on 2D TCPS proliferate, D2.0R cells encapsulated in Matrigel express a dormant phenotype (*106*). However, under hypoxic conditions, Ki67 expressions for MCF7 and MDA-MB-231 cells are consistent across 2D culture, 3D collagen gel encapsulation, and suspension cultures, suggesting that hypoxia overrides dimensionality-dependent changes in dormancy behavior (*105*).

Stiffness is a well-established cell signaling mechanism (*118*), with stiffer 2D substrates generally promoting proliferation (*119*). 2D culture of breast cancer cells on relatively soft (0.4 kPa) and stiff (4.5 kPa) hyaluronic acid substrates matches this trend, with the softer substrate promoting dormant characteristics (*120*, *121*). In 3D, stiffer gels are associated with a more dormant phenotype (*47*, *122*). Notably, the dominant in vivo site of dormancy, bone marrow, is one of the softest tissue environments (*123*). The discrepancy in the role of environmental stiffness in 2D and 3D may be because a 3D environment introduces the potentially confounding factors of cell confinement and/or environment-restricted solute transport. 3D cell confinement engages additional signaling pathways (*113*, *124*). Typical structural changes in hydrogels used to increase stiffness also decrease solute transport, potentially simulating serum deprivation or restricting cytokine access (*125*, *126*). These overlapping physical properties in 3D may require a more precise analysis of the role of stiffness in regulating breast cancer cell dormancy.

Viscoelasticity is an underinvestigated physical influence on breast cancer dormancy. Viscoelasticity is the time-dependent relaxation of stiffness under applied load, and it is present to varying extents and instructive to cells in all biological tissues (*112*). MCF7 cells cultured on viscoelastic polymer melt surfaces acquired dormant and senescent characteristics dependent on the bulk relaxation time (80 to 290 ms or elastic control), indicating a strong sensitivity to environmental viscoelasticity (*127*). To our knowledge, no other studies have directly investigated the relationship between viscoelasticity and dormancy. Given the ubiquity of viscoelasticity in biological tissues and ongoing refinements of viscoelastic biomaterials [via physically and dynamically crosslinked hydrogels (*128*, *129*)], this gap should soon be addressed.

Cellular confinement by a stiff, nondegradable environment and suspension in liquid media represent two extremes of cell expansion potential that can be investigated in vitro. Single-cell confinement in noncell-degradable environments induces dormancy within 3 days (*107*, *108*). Breast cancer cells proliferate freely in suspension (*130*) unless subject to hypoxia (*105*). Cell confinement is related to 3D stiffness but can trigger distinct mechanisms of cytoskeletal, cytoplasmic, and nuclear pressure (*113*, *124*, *131*) instead of focal adhesion–based stiffness signaling (*132*). This mechanistic distinction highlights the need for careful study investigating the effects of stiffness and confinement on breast cancer dormancy in 3D.

Overall network organization, including polymer volume fraction and ECM anisotropy, physically promotes specific cell behaviors, including breast cancer dormancy. Higher polymer weight percent PEG hydrogels (10% versus 6%) maintain lower proliferation of T47D breast cancer cells over 40 days (*60*). This study defined the environment in terms of network structure (polymer weight percent) but matches the dormancy influences of the previously discussed 3D studies since a higher weight percent corresponds to higher stiffness and a more confining cell environment. Culture on artificially introduced fibronectin has a different effect on dormancy than cell-excreted fibronectin does, and Barney *et al.* (*23*) argued that this is due to how the cells organize the fibronectin to support survival, likely by forming locally anisotropic networks. Biomaterial scientists are well-equipped to further investigate how anisotropy affects breast cancer dormancy (*114*).

Last, in vitro control of breast cancer cell seeding density and cluster size provide insight into the spatial requirements for cell-cell communication in dormancy. Increasing cell seeding density and cluster size enables escape from the dormancy-promoting influence of a softer hyaluronic acid substrate, demonstrating that dormancy is coregulated by both substrate stiffness and proximity to other breast cancer cells (*120*, *121*). Further studies with dense and sparse cocultures of breast cancer and tissue-specific cells will further clarify how cell proximity influences dormancy.

The overlapping influences of distinct physical factors in 3D in vitro environments, such as the cellular perception of 3D stiffness, confinement, and restricted solute transport, require more nuanced environments to help tease them apart. Critically, no studies have isolated or investigated the influence of hydrogel-restricted solute transport on breast cancer dormancy. 3D encapsulation in hydrogels substantially restricts the transport of large solutes to cells (*116*, *133*), which may create an unintended serum deprivation effect, passively promoting dormancy. Studies aiming to associate 3D stiffness with a specific cell behavior in vitro should include an equivalent stiffness microporous control (perhaps by using a granular hydrogel system) so that confinement and solute transport to cells are not confounding factors.

## DORMANCY TESTBEDS—NEEDS AND OPPORTUNITIES

In vitro model systems hold incredible power to parse the contributions from the ECM of the stroma and parenchyma, the cellular milieu, growth factors and cytokines, and even hypoxia and stiffness on cell and tumor dormancy. The control over a cell/tumoroid’s environment that these tunable biomaterials provide allows for reductionist studies simply not possible in vivo. These in vitro systems are far from replacing animal models, rather their maximum value is when used to hypothesis test and screen in tandem with in vivo experiments. In this section, we will detail existing dynamic and responsive biomaterials that have yet to be exploited by the dormancy field and outline needs for biomaterial innovation that could shape the future of dormancy testbeds.

**Fig. 4.**
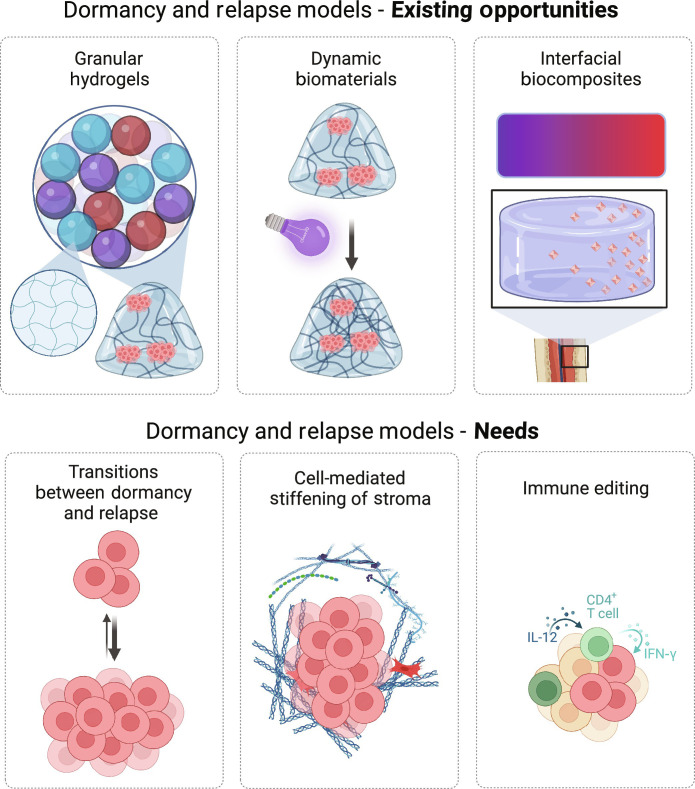
Opportunities and needs for biomaterial models of dormancy. Top: Illustrative examples of several examples of existing biomaterials technologies that exist for studies in tissue engineering, cancer models, etc. but have not yet been applied to studies in cancer dormancy. Bottom: Illustrations of technologies that are of critical need to effectively study dormancy in vitro but that do not yet exist. For example, current biomaterials do not allow to follow, in real-time, dynamic transitions between cell or tumor dormancy and relapse states, or observe the ability of immune cells to edit tumors and keep them in a dormant state. Figure created in BioRender.

As described earlier and in other reviews, there is a clear role for integrin-mediated attachment in regulating dormancy at metastatic sites (*134*). Parsing these roles is incredibly important, as even specific isoforms of individual ECM proteins could mean the difference between disease-free tissue, tumor growth, invasion, metastasis, or dormancy (*135*–*138*). Biomaterial models are perfectly suited to study the role of specific integrin engagement at metastatic sites and dynamic changes in those environments that lead to transitions between dormancy and relapse (Fig. 4). These models can unmask integrin binding sites via photo-sensitive reactions, and several external triggers (light, temperature, and even force) can be similarly used to reveal crosslinking sites (*139*–*143*). These biochemically dynamic environments have been created and applied to study tissue fibrosis and invasion at the primary tumor site (*144*, *145*), and dormancy is an obvious extension of these models. Dynamic materials—both biologically derived and from synthetic precursors—are seeing incredible attention and innovation, but few are being applied to cancer. In a recent review, our group and others highlighted the synthetic chemical strategies to create such environments and approaches to synthesize materials that stiffen, soften, swell, change shape, and more in response to a variety of external and cell-mediated triggers (*146*). These environments could represent the ECM changes that happen at secondary tissue sites coincident with tumor relapse. 

One example of such a dynamic ECM change is externally-applied force. Compressive stress is linked to stress response in breast cancer cells (*147*), leading to p38 activation, which is linked to cellular dormancy (*24*, *26*). Most commonly explored in cartilage and bone applications, tissue engineers have created several biomaterial models of dynamic compression (*148*, *149*). These models have been applied to breast cancer metastasis, linking high bone strain to reduced metastasis (*150*). Although not yet applied specifically to dormancy, the reduced overt metastases in these models could imply dormant cultures.

At the primary tumor site, stromal remodeling by local cancer-associated fibroblasts (CAFs) is a key driver of tumor growth and metastasis. Cell-mediated stiffening of the stroma at the tumor site can generate pathways of metastatic escape for epithelial cells while simultaneously prohibiting access to immune cells. Fibrous ECMs rich in collagen I regulate epithelial cell phenotype (*151*), and the nature of collagen I at the primary tumor can be classified as a “tumor-associated collagen signature,” known as TACS-1, TACS-2, and TACS-3 (*152*). In contrast, collagen III is associated with dormancy of disseminated cells (*39*). Although we do know that the local ECM is remodeled at metastatic sites, this is rarely modeled dynamically in vitro with biomaterials. Synthetic biomaterials have been designed to be largely inert to the cells embedded within them unless specific bioactive peptides or proteins have been incorporated (*87*, *153*). These materials are either macroporous or degradable by cells, via leaching techniques or incorporation of protease-cleavable crosslinks (*154*). Although degradable portions can be introduced, many of these hydrogels resist ECM protein adsorption (although some ECM proteins can be sequestered at the cell membrane) (*155*). Therefore, they soften over time during culture but do not build up a collagen-rich stroma akin to the TME during progression. A critical biomaterial advance needed to model fibrosis at the primary and metastatic tumor sites is a process for cells to deposit and retain ECM proteins with this native structure, which would determine whether this ECM remodeling is required for metastatic relapse.

How might we marry the controllability of synthetic systems with the structure and biochemical complexity of real ECM proteins? This is incredibly important, as protein structure, posttranslational modifications, and isoforms of specific proteins are all implicated in dormancy (*80*, *156*). ECM protein sequence and structure are important for reversibly binding growth factors and serving as depots for proteins and small molecules that could awaken dormant cells. Peptide-based hydrogels are one possible solution [reviewed here (*157*)], which allow cell-secreted proteins to bind, but they have not been explored in the context of breast cancer dormancy. Cell-derived ECMs are another alternative, as they are created by ECM-secreting CAFs and are modifiable by embedded cells (*158*–*160*). This concept could be extended to models of dormancy by using fibroblasts isolated from sites of metastasis that produce the ECM stroma at metastatic sites. Dormancy-relevant growth factors are released from local vasculature and then transported through the stroma, resulting in a haptotactic or chemotactic gradient. These gradients are achievable within biomaterial composites and interfacial materials (*161*, *162*) but have not been applied to metastatic sites or breast cancer dormancy studies. Creating biomimetic mechanical heterogeneity, known to be important in metastasis, across a synthetic biomaterial is another challenge. Granular hydrogels are a controllable option for creating intentionally heterogeneous materials from jammed or interconnected composite microgels (Fig. 4) (*163*, *164*). One could imagine including several microgels of varying stiffness within a broader gel or even using them as a way to release growth factors from different gels. This is a relatively new and promising technology that could be very useful to study metastasis, dormancy, and relapse in heterogeneous environments.

An increasing number of papers are revealing that metastasis occurs at an early stage. These metastatic seeds disseminate early (before a primary diagnosis), but they remain quiescent for decades (*165*). This is a dramatic transition, from a highly invasive, migratory single cell to a solitary, viable, but quiescent cell in a completely different environment. Biomaterial scientists have the unique ability to create environments that mimic primary versus metastatic sites. There is an incredible opportunity here, by combining this biomaterial design with single-cell capture or fluorescence-activated cell sorting, to move individual cells from one environment to another.

Since cells are often uniquely paired with a specific tissue site, and tissues have specific ECM proteins, growth factors, other neighboring cells, and stiffnesses, biomaterials have historically been designed in a manner that optimizes the viability and growth of specific target cells. This is philosophically in conflict with the culture of tumor cells, which can travel to, invade, and grow in a variety of distant tissues, and they are genetically (and certainly phenotypically) (*166*) heterogeneous as a result of mutations. Wonderful work has followed these genomic alterations of these tumor populations in humans and mice but not in and on biomaterials (*167*). To uncover how these ECM features may be causative or coincident with tumor evolution, at the metastatic site or elsewhere, these genomic studies need to be paired with long-term studies in and on these controllable biomaterial models, either with mutagenic cell lines or patient-derived organoids. To achieve this, biomaterial studies must be extended from days and weeks timelines to far further out. This is particularly important for dormancy, which phenomenologically occurs over timescales in real tumors over months, years, and decades. These cells remain quiescent (or tumors are growth-death rate balanced) for years. A challenge for the biomaterials community is identifying how to use in vitro systems to study a process that happens over decades. Does this require years-long experiments and therefore ultrastable materials? Can in vitro models of accelerated biological time be applied to cancer dormancy? Or does this require a more sophisticated computational abstraction between week-long experiments to multiyear phenomena?

As highlighted in Fig. 3, the vast majority of dormancy studies in vitro are limited to the highly proliferative MDA-MB-231 and the relatively nonmetastatic MCF7 cell lines. The D2 series cells are a highly effective model for proliferation versus dormancy, but they are murine. Expansion to broader sets of cell lines or even to patient-derived organoids will determine whether findings from the D2 series can be extrapolated. Going further, patient-derived organoids are a unique opportunity to understand the intersection of sex and ancestry in dormancy and metastasis. Men do get breast cancer, yet that disease is distinct from the disease in women. Several cancers that affect both sexes and span all ancestries (although potentially at different rates) exhibit dormancy. There is an increasing effort to understand sex- and ancestry-driven differences in disease (*168*–*170*). To tackle this, the field needs broader representation in cell lines and organoids.

Beyond representing the cancer cells, the cells within a dormant tumor are richly heterogeneous. These tumors contain dormant cancer cells, senescent cells (both stromal and cancerous) (*106*), immune cells (Fig. 4), blood vessels, and more. Thus far, biomaterials engineers have demonstrated their models’ abilities to capture a limited number of these cell types and/or phenotypes at a time. Yet, in tumor dormancy, these cell mixtures and phenotypes coexist, resulting in a diverse and balanced tumor-level behavior. Further, entrance into and exit from dormancy can be regulated by these other local cells., e.g., neutrophils (*171*) and lung fibroblasts (*172*). Certainly, coculture of many cells, including as cancer models, is possible and documented, but it has only seen limited applications in this way. Going further, there is likely ECM regulation of senescence and immune cell motility and exhaustion, so there is a rich opportunity to probe the multidimensional parameter space here using biomaterial models. Advanced coculture microenvironments also provide an opportunity for distinguishing between cellular and tumor dormancy, such as by seeding single cancer cells into microphysiological niches. The typical in vitro paradigm of culturing many cancer cells with shared media enables signaling between cancer cells that blurs the line between cellular and tumor dormancy.

The immune component of metastatic sites has been studied in vivo, but in vitro biomaterial systems have not yet achieved successful cocultures between cancerous cells and the immune component of the tumor (*173*). Macrophages, natural killer cells, neutrophils, and cytotoxic T cells are rarely included in in vitro models, yet they are clearly important for immune editing of metastases and maintaining tumor dormancy (Fig. 4). Immune cells can be carried over from some patient-derived organoids (*174*), but there is a need for more sophisticated biomaterial designs that include the elements required to keep these immune cells alive (*175*).

When considering adding complexity to biomaterial models such as this, there is a notable juxtaposition of competing needs. On the one hand, a major advantage of biomaterial models is the user-defined control over individual attributes: e.g., the TME stiffness, cell types included, ECM proteins, etc. Control over these individual elements is not possible with in vivo models, so there is an opportunity to test hypotheses in a reductionist manner. On the other hand, there is increasing interest in organ-on-chip or “tumor-on-chip” models which are designed to maximally represent tumor physiology (*176*). Organs-on-chip are essentially microtissues, which commonly use microfluidics to combine 3D cell cultures with physiological flow rates (and shear stresses), continuous medium replenishment, and normoxic conditions, perhaps mimicking real tissues more accurately than any other in vitro model (*176*). Initially created as single-organ systems (e.g., liver, heart, lung, etc.), recent engineering breakthroughs have linked these systems together in series and/or parallel to create interconnected multi-organ systems. These have been applied to disease modeling to understand the cytokine perturbations that lead to or exaggerate inflammation, cirrhosis, cardiovascular disease, and more. There is an immense untapped opportunity here to include tumors-on-chip within these multi-organ systems to screen for cancer drugs that kill tumors with scant side effects, to study metastasis, and to investigate transitions between dormancy and proliferation in specific organs (*177*). Although these could provide a better chance to reflect in vivo tumor biology, they are typically difficult to work with, and they are no more high-throughput than an animal model. An ideal in vitro model system would contain the complexity existent in vivo yet would be simultaneously high throughput, economical, and reproducible.

## CONCLUSIONS

Breast cancer dormancy is a multifaceted aspect of breast cancer biology and treatment that requires more detailed studies to understand the associated mechanisms and viable treatment approaches. While in vivo studies remain critical for validating preclinical hypotheses and capturing the complexities of cancer treatment, in vitro models provide unique opportunities to isolate mechanisms, trigger specific cell responses, and extract more information from cancer cell lines and patient-derived samples. Specifically, in vitro models are needed to untangle the many overlapping cell-environment interactions that govern advanced, responsive cell behaviors such as dormancy and reactivation. However, in vitro modeling still requires further development to reach its full potential in many respects, especially the long-term culture needed for the development of cell-environment feedback cycles. Future research that applies cutting-edge biomaterial design to solving persistent questions in breast cancer dormancy will reveal mechanisms and generate biological hypotheses that will ultimately contribute to effective clinical treatment of dormant breast cancer.

## CITATIONS DIVERSITY STATEMENT

Recent work in several fields of science has identified a bias in citation practices such that papers from women and other minority scholars are under-cited relative to the number of such papers in the field (*178*–*185*). Here, we sought to proactively consider choosing references that reflect the diversity of the field in thought, form of contribution, gender, race, ethnicity, and other factors. First, we obtained the predicted gender of the first and last authors of each reference by using databases that store the probability of a first name being carried by a woman (*180*, *186*). By this measure and excluding self-citations to the first and last authors of our current paper, our references contain 18.81% woman (first)/woman (last), 14.45% man/woman, 32.43% woman/man, and 34.31% man/man. This method is limited in that (i) names, pronouns, and social media profiles used to construct the databases may not, in every case, be indicative of gender identity, and (ii) it cannot account for intersex, nonbinary, or transgender people. Second, we obtained predicted racial/ethnic category of the first and last authors of each reference by databases that store the probability of a first and last name being carried by an author of color (*187*, *188*). By this measure (and excluding self-citations), our references contain 19.4% author of color (first)/author of color(last), 15.73% white author/author of color, 25.37% author of color/white author, and 39.50% white author/white author. This method is limited in that (i) names and Florida Voter Data to make the predictions may not be indicative of racial/ethnic identity, and (ii) it cannot account for Indigenous and mixed-race authors or those who may face differential biases due to the ambiguous racialization or ethnicization of their names. We look forward to future work that could help us to better understand how to support equitable practices in science.
